# Topical lipoic acid choline ester eye drop for improvement of near visual acuity in subjects with presbyopia: a safety and preliminary efficacy trial

**DOI:** 10.1038/s41433-020-01391-z

**Published:** 2021-01-29

**Authors:** Michael S. Korenfeld, Stella M. Robertson, Jerry M. Stein, David G. Evans, Steven H. Rauchman, Kenneth N. Sall, Subha Venkataraman, Bee-Lian Chen, Mark Wuttke, William Burns

**Affiliations:** 1 Comprehensive Eye Care Center Ltd, 901 East Third Street, Washington, MO 63090 USA; 2 Arrochar Consulting, LLC, 7045 Shadow Creek Court, Fort Worth, TX 76132 USA; 3 Summer Creek Consulting, LLC, 8101 Rain Dance Trail, Fort Worth, TX 76123 USA; 4 Total Eye Care, PA, 6060 Primacy Parkway, Memphis, TN 38119 USA; 5 North Valley Eye Medical Group, 11550 Indian Hills Road, Mission Hill, CA 91345 USA; 6 Sall Research Medical Centre, 11423 87th Street, Artesia, CA 90701 USA; 7 Novartis Pharmaceutical Corporation, 1 Health Plaza, East Hanover, NJ 07936 USA; 8 Novartis Pharma AG, Basel, Switzerland; 9 Encore Vision, Inc., 1120 South Freeway, Suite 118, Fort Worth, TX 76104 USA

**Keywords:** Outcomes research, Prognosis

## Abstract

**Objectives:**

This study evaluated the safety of topical lipoic acid choline ester (UNR844, 1.5%) ophthalmic solution and its efficacy in improving distance-corrected near visual acuity (DCNVA) in subjects with presbyopia.

**Subjects and methods:**

This was a prospective, randomized, double-masked, and multicentre clinical trial. Subjects with a diagnosis of presbyopia (*n* = 75) were randomized 2:1 to UNR844 or placebo. On days 1–7, all subjects were dosed unilaterally (twice a day, b.i.d.) in their non-dominant eye to ensure safety and tolerability prior to days 8–91 when dosing was changed to bilateral (b.i.d.). Clinical assessments, including DCNVA and adverse events (AEs), were recorded at each study visit. Patients who completed the study were recruited into a non-interventional follow-up study that monitored them until 7 months after their final UNR844 exposure. The primary endpoints were safety and the mean change in DCNVA from baseline in the study eye.

**Results:**

UNR844 administration (*n* = 50) produced no safety concerns and was well-tolerated, with no clinically-relevant changes in best-corrected distance visual acuity, pupil size, intraocular pressure, or discontinuations due to adverse events. DCNVA improved in the study eye in the UNR844 group compared to placebo during the 91 days of treatment [UNR844 vs. placebo, mean change in LogMAR (SD); −0.159 (0.120) vs. −0.079 (0.116)]. Bilateral DCNVA improved, with 53.1% UNR844 vs. 21.7% placebo subjects gaining ≥10 letters. Improvements in DCNVA were sustained at 5 and 7 months after UNR844 dosing ceased.

**Conclusions:**

These results support further development of UNR844 ophthalmic solution for the treatment of presbyopia.

## Introduction

Presbyopia is natural, age-related decline in near vision resulting from a progressive decrease in the accommodation mechanism of the eye. This progressive decline becomes apparent from about 40 years of age as it affects the individual’s ability to focus on near objects and to perform near visual tasks. Without optical correction, presbyopia can have multiple effects on vision quality and quality of life [1]. Presbyopia is a global problem, with an estimated 1.3 billion presbyopic patients worldwide. The prevalence is projected to rise to 1.8 billion people by 2050 as populations’ age [2].

Currently, there are no approved pharmacological therapies designed to treat presbyopia. External ocular lenses, such as spectacles or contact lenses, are typically used to correct vision [1]. Ocular surgery and surgical devices, such as corneal inlays [3], multifocal or extended depth of field (EDOF) intra-ocular lenses [4–6] and refractive laser [7, 8] can also be used as treatment options, but these are invasive procedures, they are expensive and some treatments can only treat one eye (monovision) with a loss of optical summation from binocular vision [1, 3–8]. Access to spectacles is also problematic in developing countries, where 94% of presbyopic subjects reside [2]. There is a clear need for an effective, safe and tractable disease-modifying treatment.

Presbyopia is the result of a complex degeneration of the ciliary body, vitreous body and the crystalline lens. The degenerative changes of the crystalline lens are postulated to occur due to changes in the elasticity of the lens capsule and its contents [9–12], and in the overall lens size and shape [13]. A major contributing factor to the loss of lens elasticity is thought to be an age-dependent increase in the formation of disulfide bonds between crystalline lens proteins due to oxidative stress [9, 14–17]. Normal accommodation depends on the displacement of cytosol in the lens fiber cells to increase the refractive index of the lens [18, 19]. As the lens continues to grow with advancing age, there are insufficient reducing enzymes servicing the peri-nuclear cell layers to maintain unbounded proteins and the free flow of cytosol [15]. This leads to a loss of lens elasticity and dynamic refractive power during accommodation.

Lipoic acid (LA) is an antioxidant shown to chemically reduce lens disulfide bonds. In preclinical studies, topical LA dose-dependently increased lens elasticity in vitro [20]. LA is produced in the mitochondria of all cells. LA has also demonstrated safety but limited ocular penetration, due to its lipid solubility, following topical administration to the eye in single-dose studies [21]. UNR844 (formerly known as EV06) is a lipoic acid choline ester and a potential first-in-class disease-modifying topical treatment for presbyopia. It is designed to be a pro-drug. Linking LA to choline increases its corneal penetration and enables a therapeutic dose of LA to be achieved in the aqueous humor. LA is taken up into lens fiber cells where it is metabolized by oxidoreductases to the active species, dihydrolipoic acid (DHLA), which reduces disulfide bonds between lens proteins, putatively improving the dynamic refractive power of the lens during accommodation and improving near visual acuity [20]. In this multicentre, Phase 1/2 study, the aim was to investigate the safety and efficacy of UNR844 in improving distance-corrected near visual acuity (DCNVA) in subjects with presbyopia.

## Methods

This was a prospective, randomized, double-masked, placebo-controlled, multicentre study to evaluate the safety and efficacy of UNR844 on DCNVA in subjects with a diagnosis of presbyopia (ClinicalTrials.gov identifier NCT02516306). The study protocol was approved by the Sterling Institutional Review Board and complied with the ethical standards defined by the Declaration of Helsinki and Good Clinical Practice. The study was conducted at four sites in the United States (Evans, Korenfeld, Rauchman, Sall).

### Study participants

Subjects included in the study were 45–55 years of age, had monocular DCNVA worse than 20/40 in each eye, and a best-corrected distance visual acuity (BCDVA) of 20/20 or better in each eye. Concomitant hyperopia or myopia was allowed, as long as the manifest refraction spherical equivalent was between or equal to +4.0 dioptres (D) and −4.0 D and there was a difference of ≤0.5 D between manifest and cycloplegic refraction spherical equivalents. Subjects with a contraindication to pupil dilation, untreated occludable angles in either eye, a pupillary diameter <2.5 mm prior to dilation, insufficient dilation, unequal pupil diameters, congenital ocular malformations, or with ocular hypertension and/or glaucoma, were excluded from the study (see Supplementary materials for further details). All subjects underwent an informed consent process prior to enrollment and signed informed consent and Health Insurance Portability and Accountability forms.

### Study design

Enrolled participants (*n* = 75) were randomized in a 2:1 ratio using a web-based interactive response system to receive UNR844 (lipoic acid choline ester, 1.5%; Encore Vision, Inc.) or ophthalmic placebo solution (vehicle) (Fig. S1a). This was a Phase 1/2 exploratory clinical study and no formal hypothesis testing was performed. A sample size of 72 subjects was determined based on establishing a reasonable number of subjects to provide adequate safety and efficacy information to proceed to the next phase of clinical development. No formal power calculations were used to determine sample size.

Randomization was stratified into two sub-groups at visit two, with one having DCNVA of better than 20/80 and the other DCNVA that was equal to or worse than 20/80, as pre-specified in the SAP in order to explore possible treatment effects due to differences in baseline DCNVA in presbyopes. Since this was a first-in-human study, the non-dominant eye (as determined at the screening visit by the subject’s perception of a distant object relative to their hands using the triangle-miles [22] or finger-porta methods [23]) was pre-designated the study eye for initial monocular safety and tolerability evaluation. UNR844 or placebo ophthalmic solutions were dosed in two treatment periods. On days 1–7, all subjects were dosed unilaterally b.i.d. in their non-dominant eye (the study eye) and then from days 8 to 91 dosing was bilateral (b.i.d.; Fig. S1b). Doses were self-administered at home, except for the first dose, which was self-administered in the clinic under supervision.

### Outcomes and assessments

Clinical assessments were recorded at each study visit in each eye, unless otherwise stated. The primary outcome was safety, assessed by adverse events (AE; coded using MedDRA Version 18.0 or higher), acute comfort assessments and ocular safety assessments, which included BCDVA (LogMAR and Snellen equivalent), intraocular pressure (IOP; mmHg), slit-lamp biomicroscopy and fundus examination. Acute comfort assessments using a visual analog scale (0, very comfortable; 10, very uncomfortable) were performed in the clinic on Day 1 (baseline) immediately prior to and after the first instillation, followed by self-assessments on the day before each visit to the clinic.

The primary and secondary efficacy endpoints were, respectively, mean change in DCNVA and the proportion of subjects with a gain of ≥10 letters in DCNVA from days 1 to 91 in the study eye. Non-study eye and bilateral vision assessments were also measured, although they were not the primary and secondary efficacy endpoints. DCNVA was measured at 40 cm in a room with controlled lighting conditions of 8–15 lux, using an ETDRS LogMAR chart provided by the M&S technologies clinical trial system (CTS), which is a validated vision testing technology. Snellen equivalents were calculated by the CTS instrument with subjects corrected for any distance refractive errors. The M&S CTS system automates vision testing, providing a randomized ETDRS LogMAR chart with standardized lighting for each visual assessment, which helps to minimize memorization of the chart by the subject.

Exploratory endpoints were change from baseline in the manifest and cycloplegic distance refraction, non-dilated pupillary diameter of each eye and subjective (defocus curve) and objective accommodative amplitude in the study eye. At each visit, manifest refraction of sphere (D), cylinder (D), axis (degrees) and spherical equivalent (D) were recorded. The M&S CTS system was utilized for visual assessment testing for refractive error (sphere, cylinder, manifest refraction), BCDVA, DCNVA assessed at 40 cm and defocus curve testing. (See Supplementary materials for additional details).

### Follow-up study

Patients who completed the 91-day interventional study phase (*n* = 72) were optionally recruited (*n* = 52) for a non-interventional follow-up study to evaluate long-term vision performance ~5 months (day 241) and 7 months (day 301) after final dosing with UNR844 (Fig. S1a, b). All assessments were performed by site personnel masked to the initial treatment assignment.

### Statistical analysis

Statistical analysis used the full analysis set (FAS) and was performed using the study eye in all primary endpoint analyses (last observation carried forward [LOCF]) per the statistical analysis plan (SAP). Continuous variables were summarized by descriptive statistics (sample size, mean, standard deviation, median) while discrete variables were summarized by frequencies and percentages.

Ad hoc statistical analysis used the FAS, with non-LOCF data. Independent *t*-tests for normal continuous data and Pearson’s chi-squared or Fisher’s exact tests for categorical data were conducted. All *p* values associated with the ad hoc analysis are nominal with two-sided significance level set at 0.05. Calculations on each time point were performed on an *n* by day basis (subjects present and observed at the visit day; no carry forward). Day 1 and day 91 comparisons included only subjects that completed the study (placebo, *n* = 23; UNR844, *n* = 49). For treatment group comparisons, two analysis of covariance (ANCOVA) models and Fisher’s exact test were included for the primary and secondary efficacy endpoints, respectively. Statistical analysis for the follow-up, observational study used the FAS and was performed using the study eye and both eyes (bilateral) in all primary endpoint analyses (non-LOCF, no missing data imputation), per the SAP.

## Results

### Subject demographics and baseline characteristics

A total of 75 subjects were randomized of whom 72 subjects completed the study. Two subjects discontinued due to non-compliance and the third withdrew from the study (Fig. S1a).

Baseline demographics were well-balanced between the UNR844 treated groups and placebo in the overall and stratified cohorts. The overall mean age was similar between the UNR844 treated group and placebo (50.1 and 51.4 years, respectively), with most subjects being female (70.7%). There were no major differences in race or ethnicity. The majority of subjects were emmetropic (51 subjects; 68%), compared with 15 (20%) myopes and 9 (12%) hyperopic subjects, with a comparable distribution across study groups. Baseline DCNVA mean LogMAR [SD] was similar in the study eye and non-study eye (0.507 [0. 11]; 0.507 [0.10] UNR844 and 0.500 [0.10]; 0.509 [0.10] placebo groups) and slightly better in bilateral vision (0.397 [0.10] UNR844 vs. 0.408 [0.12] placebo), illustrating ocular summation, in which bilateral vision is improved over monocular vision due to the fusion in forming human binocular vision by the brain from two monocular views of an object [24]. Baseline demographics and refractive status were also well balanced during the follow-up phase and generally similar to the interventional phase of the study (Table 1).

**Table 1:** Demographics and baseline characteristics (FAS).

Intervention study	Observational study							
	DCNVA Better than 20/80 (*n* = 50)		DCNVA 20/80 or worse (*n* = 25)		Overall (*n* = 75)		Overall (follow-up cohort) (*n* = 52)	
	Placebo (*n* = 17)	UNR844 (*n* = 33)	Placebo (*n* = 8)	UNR844 (*n* = 17)	Placebo (*n* = 25)	UNR844 (*n* = 50)	Placebo (*n* = 18)	UNR844 (*n* = 34)
Age (years)								
Mean (SD)	51.2 (3.1)	49.3 (3.4)	51.8 (2.7)	51.6 (2.3)	51.4 (3.0)	50.1 (3.2)	52.6 (3.0)	50.9 (3.4)
Median	52.0	49.0	52.0	52.0	52.0	50.0	54.0	51.0
Min Max	45.0, 55.0	45.0, 55.0	48.0, 55.0	47.0, 55.0	45.0, 55.0	45.0 55.0	46.0 56.0	45.0 56.0
Gender								
Female, *n* (%)	12 (70.6%)	23 (69.7%)	8 (100.0%)	10 (58.8%)	20 (80.0%)	33 (66.0%)	14 (77.8%)	24 (79.4%)
Male, *n* (%)	5 (29.4%)	10 (30.3%)	0 (0%)	7 (41.2%)	5 (20.0%)	17 (34.0%)	4 (22.2%)	10 (29.4%)
Race								
White, *n* (%)	11 (64.7%)	21 (63.6%)	7 (87.5)	14 (82.4%)	18 (72.0%)	35 (70.0%)	13 (72.2%)	27 (79.4%)
Black or African American	6 (35.3%)	12 (36.4%)	1 (12.5%)	3 (17.6%)	7 (28.0%)	15 (30.0%)	5 (27.8%)	7 (20.6%)
Ethnicity								
Hispanic or Latino, (%)	4 (23.5%)	7 (21.2%)	1 (12.5%)	9 (52.9%)	5 (20.0%)	16 (32.0%)	3 (16.7%)	12 (38.2%)
Not Hispanic or Latino, *n* (%)	13 (76.5%)	26 (78.8%)	7 (87.5%)	8 (47.1%)	20 (80.0%)	34 (68.0%)	15 (83.3%)	21 (61.8%)
Refractive status								
Myopes	4 (23.5%)	7 (21.2%)	2 (25.0%)	2 (11.8%)	6 (24.0%)	9 (18.0%)	4 (22.2%)	7 (20.6%)
Emmetropes	11 (64.7%)	22 (66.7%)	6 (75.0%)	12 (70.6%)	17 (68.0%)	34 (68.0%)	12 (66.7%)	21 (61.8%)
Hyperopes	2 (11.8%)	4 (12.1%)	0 (0.0%)	3 (17.6%)	2 (8.0%)	7 (14.0%)	2 (11.1%)	6 (17.6%)
Baseline DCNVA mean LogMAR (SD)								
Study eye	0.445 (0.05)	0.444 (0.05)	0.617 (0.06)	0.629 (0.07)	0.500 (0.10)	0.507 (0.11)	0.489 (0.08)	0.507 (0.11)
Non-study eye	0.459 (0.06)	0.467 (0.09)	0.615 (0.10)	0.585 (0.08)	0.509 (0.10)	0.507 (0.10)	0.490 (0.09)	0.506 (0.09)
Bilateral	0.358 (0.09)	0.348 (0.08)	0.515 (0.10)	0.492 (0.07)	0.408 (0.12)	0.397 (0.10)	0.406 (0.11)	0.408 (0.10)

Any protocol deviations including prior and concomitant use of prescription and over-the-counter medications were judged not to have any impact on the safety or efficacy results.

### Efficacy outcomes

**Fig. 1:**
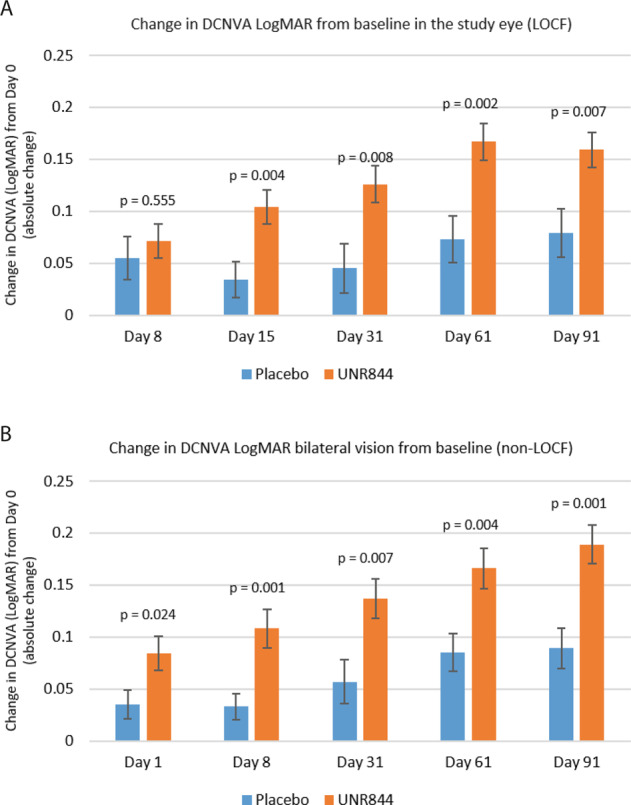
Improved near vision over time change in DCNVA. **A** Improved near vision over time (Day 8–91) change in DCNVA LogMAR from baseline in the *study eye.* **B** Improved near vision over time (Day 8–91) change in DCNVA LogMAR *bilateral vision* from baseline (non-LOCF).

The study showed a progressive improvement in near vision with UNR844 treatment during the 91 days of dosing. From baseline to day 91, UNR844-treated subjects demonstrated an improved DCNVA in mean change (SD) LogMAR of −0.159 (0.120) compared with −0.079 (0.116) for the placebo group in the study eye (*p* = 0.007; Fig. 1a). A higher percentage of subjects gained ≥10 letters in the study eye from baseline to day 91 in the UNR844 vs. the placebo group, although this was not statistically significant (37% vs. 17%; Fig. S2a). ANCOVA models determined that strata or baseline values did not contribute to treatment group differences.

The primary efficacy end point was the change in DCNVA (LogMAR) in the study eye. Since subjects assigned use of UNR844 were treated in both the study and non-study eye from day 8 to 91, an analysis of the bilateral vision was performed. Ad hoc bilateral data supported findings in the study eye, with an improved bilateral DCNVA mean change of −0.189 vs. −0.089 LogMAR units from days 1 to 91 for UNR844 vs. placebo, respectively (*p* = 0.001) (Fig. 1b; Table S1). The number of subjects with a gain of ≥10 letters in bilateral near vision was consistently higher from days 1 to 91 (53.1% vs. 21.7% in UNR844 vs. placebo-treated subjects; *p* = 0.021) (Fig. S2b). Overall, the proportion of subjects gaining 1, 2, and 3 lines of vision was greater for the UNR844 than placebo treated groups (Table S2). Furthermore, 82.0% of subjects completed the study with better than 20/40 (bilateral) vision when treated with UNR844, compared with 48.0% treated with placebo. After the study completed, it was noted that 29.3% of subjects entered the study with baseline bilateral DCNVA of 20/40 or better (although monocular DCNVA was worse than 20/40 in each eye per the inclusion criteria). This was true in 30.0% (15/50) of the UNR844 group and 28.0% (7/25) of the placebo group. Subsequent analysis of the subset that excluded presbyopic subjects that entered the study with 20/40 or better bilateral DCNVA, found an improvement in bilateral DCNVA from baseline to day 91 for the UNR844 group vs. placebo (−0.198 vs. −0.099 DCNVA LogMAR, *p* = 0.004; Table S1). In this subset, 74.3% (26/35) of subjects treated with UNR844 as compared to 33.3% (6/18) of subjects treated with placebo had 20/40 or better bilateral DCNVA at day 91 (*p* = 0.012).

### Efficacy outcomes at 5- and 7-month follow-up

**Fig. 2:**
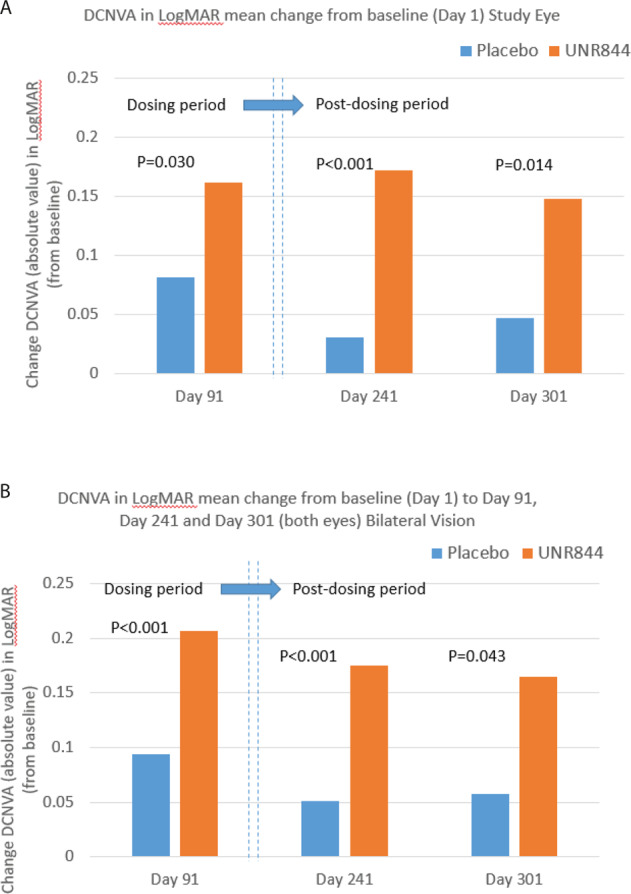
DCNVA mean change from baseline (Day 1) to Day 91, Day 241 and Day 301. **A** DCNVA in LogMAR mean change from baseline (Day 1) to Day 91, Day 241, and Day 301 (follow-up data) in the *study eye*. **B** DCNVA in LogMAR mean change from baseline (Day 1) to Day 91, Day 241, and Day 301 (follow-up data) in *bilateral vision*.

Improvements in DCNVA with UNR844 vs. placebo treatment in the study eye from baseline were sustained at 5 and 7 months after the final dosing with UNR844 (mean change of −0.172 vs. −0.031 LogMAR for days 1–241; and −0.148 vs. −0.047 LogMAR for days 1–301; *p* < 0.001 and *p* = 0.014, respectively; Fig. 2a). The percentage of subjects with a gain of ≥10 letters in the study eye from baseline to follow-up visits day 241 and day 301 was higher for UNR844 compared to placebo treated patients (45.5% vs. 11.8% at day 241 and 42.4% vs. 16.7% at day 301) (Fig. S3). ANCOVA sensitivity analysis determined that baseline differences or strata did not affect the outcome.

Findings for bilateral data supported the study eye data with an improvement in DCNVA from baseline to day 241 and day 301 for the UNR844 vs. placebo group (Fig. 2b and Table S1). A higher percentage of subjects reported a bilateral gain of ≥10 letters from baseline at day 241 (42.4% vs. 11.8% for UNR844 vs. placebo, *p* = 0.053) and day 301 (39.4% vs. 5.6% for UNR844 vs. placebo, *p* = 0.010). There was a sustained bilateral visual improvement of ≥1 line of vision at day 301 in 66.7% of subjects treated with UNR844 vs. 50.0% for placebo.

**Fig. 3:**
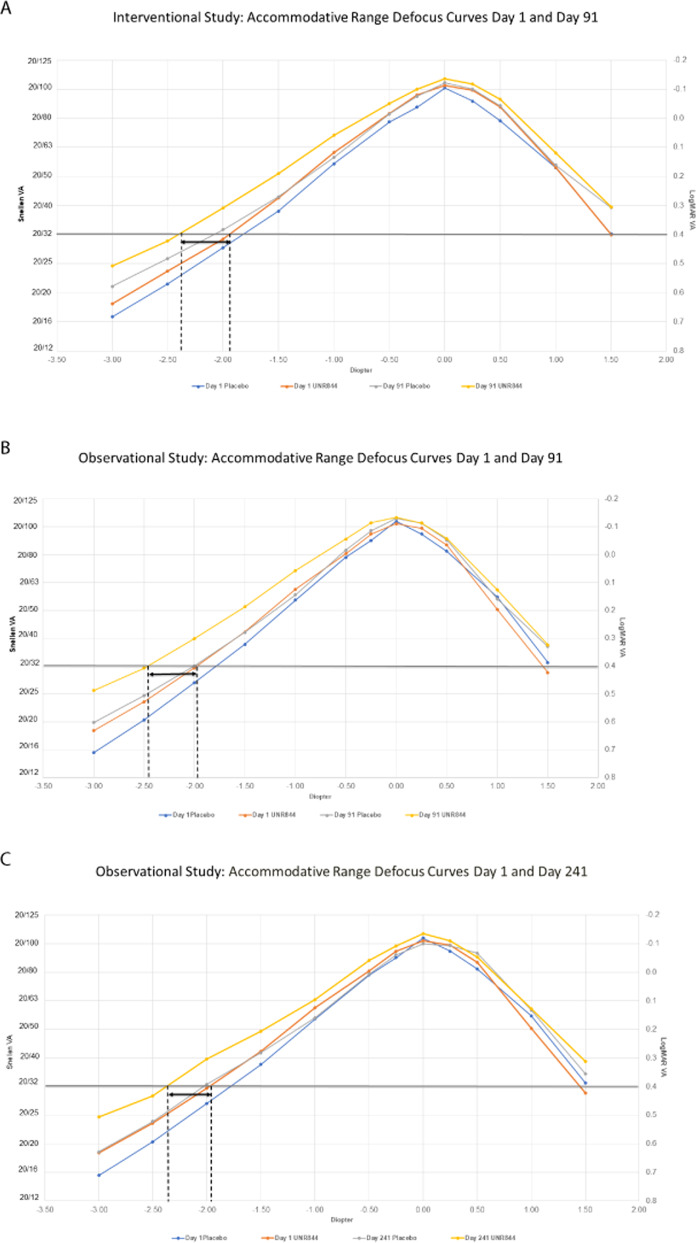
Accommodative range defocus curves day 1 to day 241 in the study eye (non-LOCF). Accommodative range defocus curves day 1 to day 241 in the *study eye* (non-LOCF). **A** Interventional Study [*n* = 75]: accommodative range defocus curves day 1 and day 91 in the *study eye* (non-LOCF). **B** Observational Study Cohort [*n* = 52]: accommodative range defocus curves day 1 and day 91 in the *study eye* (non-LOCF). **C** Observational Study Cohort [*n* = 52]: accommodative range defocus curves day 1 and day 241 in the *study eye* (non-LOCF).

No clinically meaningful differences were observed in manifest refraction, a measurement of distance vision, from baseline or last dose (Day 91) (Table S3) to day 241 or day 301 in the study eye. Defocus curve data support the sustained maintenance of improvements in near vision in the study eye from UNR844 treatment compared to placebo at day 91 (Fig. 3a interventional cohort, *n* = 75; Fig. 3b observational cohort, *n* = 52). UNR844 treatment resulted in a gain of about 0.5 D in accommodative amplitude from baseline to day 91, which continued post-treatment in the follow-up phase, with a slight decline at day 241 (Fig. 3c), to 0.25 D by day 301 (Fig. S4).

### Adverse events

#### Treatment emergent adverse events (TEAEs)

A low number of TEAEs suggests UNR844 is well-tolerated and raises no safety concerns. Overall, 26/50 (52%) UNR844 and 10/25 (40%) placebo-treated subjects experienced TEAEs (Table 2). In 11/50 (22.0%) of the UNR844 group and 3/25 (12.0%) of the placebo group, the TEAES were considered to be study drug-related. All reported TEAEs were mild or moderate in intensity, with the exception of one placebo subject with a herniated disc and one UNR844 subject with a ruptured tendon of the right finger. Both were unrelated to treatment. There were no severe TEAEs and no deaths during the study, and no subjects discontinued treatment due to adverse events, safety concerns, or tolerability effects.

**Table 2:** Treatment emergent adverse events.

	Placebo (*n* = 25)	UNR844 (*n* = 50)
Subjects with any TEAE	10 (40.0%)	26 (52.0%)
Subjects with marked/severe TEAE	1 (4.0%)	1 (2.0%)
Subjects with any study drug related TEAE	3 (12.0%)	11 (22.0%)
Subjects with any study drug related serious TEAE	0 (0.0%)	0 (0.0%)
Subjects with outcome of death	0 (0.0%)	0 (0.0%)
Subjects with any TEAE leading to study drug discontinuation	0 (0.0%)	0 (0.0%)
All Ocular TEAEs		
Asthenopia	0 (0.0%)	2 (4.0%)
Blepharitis	0 (0.0%)	1 (2.0%)
Conjunctival hyperaemia	2 (8.0%)	0 (0.0%)
Eye irritation	0 (0.0%)	3 (6.0%)
Eye pruritus	0 (0.0%)	2 (4.0%)
Eyelid oedema	1 (4.0%)	0 (0.0%)
Foreign body sensation	0 (0.0%)	2 (4.0%)
Ocular hyperaemia	0 (0.0%)	2 (4.0%)
Photophobia	0 (0.0%)	1 (2.0%)
Vision blurred	0 (0.0%)	1 (2.0%)
Instillation site irritation	0 (0.0%)	2 (4.0%)
Instillation site pain	1 (4.0%)	3 (6.0%)
Hyperaemia (vascular disorders)	0 (0.0%)	1 (2.0%)
Ocular TEAEs related to study drug		
Subjects with at least one ocular TEAE related to study drug	3 (12.0%)	8 (16.0%)
Asthenopia	0 (0.0%)	2 (4.0%)
Blepharitis	0 (0.0%)	1 (2.0%)
Conjunctival hyperaemia	2 (8.0%)	0 (0.0%)
Eye irritation	0 (0.0%)	2 (4.0%)
Eye pruritus	0 (0.0%)	2 (4.0%)
Foreign body sensation	0 (0.0%)	2 (4.0%)
Ocular hyperaemia	0 (0.0%)	1 (2.0%)
Vision blurred	0 (0.0%)	1 (2.0%)
Instillation site irritation	0 (0.0%)	2 (4.0%)
Instillation site pain	1 (4.0%)	3 (6.0%)
Hyperaemia (vascular disorders)	0 (0.0%)	1 (2.0%)
Non-ocular TEAEs related to study drug		
Subjects with at least one non-ocular TEAE related to study drug	1 (4.0%)	8 (16%)
Upper respiratory tract infection	0 (0.0%)	1 (2.0%)
Dysgeusia	0 (0.0%)	7 (14.0%)
Headache	0 (0.0%)	2 (4.0%)
Somnolence	1 (4.0%)	0 (0.0%)
Throat irritation	0 (0.0%)	1 (2.0%)

Ocular disorders were one of the most common TEAEs and considered treatment-related in 16% (8/50) and 12.0% (3/25) of subjects in the UNR844 and placebo treated groups, respectively. The most common of these related to study drug instillation site pain (6.0% vs. 4.0% for UNR844 vs. placebo, respectively). In the UNR844 group only, 4.0% of subjects reported eye irritation, asthenopia, eye pruritus, or foreign body sensation. Only conjunctival hyperaemia was found more commonly in placebo treated subjects (8.0%). Conjunctival hyperaemia was not reported in UNR844 treated patients.

A total of 22/50 (44.0%) and 7/25 (28.0%) of UNR844 and placebo treated subjects reported at least one non-ocular TEAE, with dysgeusia (14.0%) and headache (8.0%) as the most commonly reported TEAEs in the UNR844 group; no cases of either TEAE were found in placebo treated subjects.

#### Ocular safety

There were no clinically meaningful changes in the study eye from baseline to day 91 in either treatment group in non-dilated pupil diameter (Fig. S5), ocular comfort, IOP, or distance vision (Table S4), slit-lamp biomicroscopy (Table S5) or fundus findings (Table S6). (See Supplementary materials).

#### Adverse event outcomes at 5- and 7-month follow-up

At the end of the interventional phase of the study, five subjects reporting seven ongoing AEs entered the follow-up phase. During follow-up, no adverse events related to study procedures were reported, and all ongoing AEs resolved during the extension phase. As in the interventional study phase, there were no clinically significant changes compared to baseline in the study eye in BCDVA, or any significant changes in IOP measurements, with all values well within normal range across the study groups. In addition, no significant changes in the incidence of positive slit-lamp biomicroscopy findings were identified across groups.

## Discussion

UNR844 showed encouraging results for improving near vision that is reduced by presbyopia. The topical ocular unilateral and bilateral administration of UNR844 ophthalmic solution (b.i.d.) raised no safety concerns and was well-tolerated. Statistically significant improvements in DCNVA occurred as early as day 8 and were not associated with any deleterious effects on the eye, systemic complications, or degradations of distance vision. Additional exploratory outcomes supported this efficacy result, with clinically significant improvements in near vision (such as percentage of subjects gaining ≥2 lines or ≥10 letters DCNVA, or percentage with 20/40 or better DCNVA), or regardless of the analysis chosen (study eye, non-study eye, both eyes, strata, and exclusion subjects). Similar improvements in DCNVA were observed in both strata (those individuals with baseline DCNVA 20/80 or worse and those with baseline DCNVA better than 20/80) indicating that the treatment was effective regardless of the degree of presbyopia present in this sample of subjects.

An important aim in treating presbyopia is to improve binocular vision. In this study, ad hoc analysis of bilateral vision was deemed appropriate since subjects were dosed bilaterally from day 8 for the duration of the 91-day study. Ad hoc analysis of bilateral vision supported the main outcomes observed in the study eye. Bilateral DCNVA was statistically and clinically improved with bilateral dosing of UNR844. Bilateral vision maximizes stereopsis and optical summation, which is clinically relevant since both eyes will be dosed in clinical practice [24]. The determination of the non-dominant eye as the study eye was strictly done for safety reasons in this first-in-humans trial; this was the eye that received initial treatment (days 1–7). Although it is common to assign near vision optics to the non-dominant eye in “mono-vision” strategies with contact lenses and monofocal intraocular lenses, there was no reason to believe that the non-dominant eye would respond differently from the dominant eye when dosed with the active drug [25]. Evaluations of each eye were done separately to ensure that the drug produced a comparable effect in both eyes. It did. Because both eyes were dosed with the active drug, both had comparable monocular visual performance, and the fact that human vision is structured around binocularity, evaluations of binocular performance of the active drug were performed. As would be expected from binocular vs. monocular physiology, the visual performance of subjects receiving the active drug tested better binocularly than monocularly, although both improved.

In the observational follow-up study, the improvements in both monocular and binocular near vision were maintained, with the clinically significant improvements in near vision (DCNVA LogMAR) present after 3 months of UNR844 dosing being sustained for 5 and 7 months after cessation of treatment. The follow-up study subjects were similar in their demographic profile to that of the interventional phase of the study, lending validity to the results from the follow-up observations. In the follow-up study, UNR844 and placebo cohorts might have been enriched slightly, as both showed a slightly higher treatment effect at day 91 in bilateral vision compared to their respective original interventional groups (mean change in bilateral DCNVA LogMAR from day 1 to day 91, last dose for UNR844 was −0.207 for the follow-up cohort and −0.189 for the interventional group; placebo was −0.094 vs. −0.089 bilateral DCNVA LogMAR, respectively).

Data from the non-interventional study suggest that the improvements in near vision attributed to treatment with UNR844 weakened when dosing ended. A gradual decline was seen in DCNVA LogMAR values, defocus curves, and the proportion of subjects with gains ≥10 letters when comparing day 91 to day 241 and day 301. This may be the result of relentless time-dependent lens oxidation after dosing with the anti-oxidant was terminated [15, 26, 27].

Treatment with UNR844 ameliorated DCNVA changes and preserved distance vision, without impacting IOP or altering pupil size. The lack of effect on pupil size also confirms that the mechanism of action of UNR844 does not rely on the “pinhole effect”, as is seen with drugs that generate their therapeutic benefit by simply reversibly constricting the pupil for short periods of time to enhance the depth of focus. Instead, the mechanism of action for UNR844 is directed at the pathologic changes that the crystalline lens experiences with time in an oxidizing environment. These data support preclinical observations [20] and the treatment concept of using a drug to reduce disulfide bonds to allow greater cytosol displacement during accommodation to restore lens elasticity and dynamic refractive power [12, 15, 18, 28, 29]. Since treatment with UNR844 is thought to alter the durometer of the crystalline lens as the disulfide bonds are reduced, it was theoretically possible that the lens could change its gross shape and curvature as it became softer. The distance vision, manifest refraction, and cycloplegic refraction were stable during the study. This demonstrates that UNR844 is clinically safe and is only altering the dynamic characteristics of the crystalline lens and not it’s resting state characteristics.

The current study was well designed and executed, utilized accepted vision methodology [30], and included an appropriate study population compliant with treatment that was representative of target subjects. The rate of subject discontinuations was low, thus unlikely to impact study results, and no subject discontinued therapy due to an adverse event, confirming the tolerability of the agent. During the 3-month interventional study, the number of doses applied was >97% of the maximum dosing expected.

Similar numbers of subjects in both groups reported protocol deviations, such as missing 1–4 consecutive daily doses or concomitant use of medications associated with blurred vision. Protocol deviations were considered more likely to negatively than positively bias the results.

Although the number of subjects enrolled in the study was relatively small, the population was sufficient to indicate preliminary efficacy and identify any safety concerns. A relatively good response was observed in the placebo group, which is not unexpected given the subjective nature of the DCNVA assessments. Further study will be required to fully establish the clinical benefits/risk-benefit profile of UNR844 on presbyopia.

This study demonstrates that UNR844 has the potential to be a first-in-class disease modifying pharmacological therapy for presbyopia, for which there are currently no approved pharmacological treatment interventions. There is a key unmet need for a convenient and safe topical ocular treatment option for presbyopia. Phase 1/2 results from this study show UNR844 to be a well-tolerated, effective pharmacological intervention for presbyopia and support the further development of this therapeutic approach.

### Summary

#### What was known before

- Currently, there are no approved pharmacological therapies designed to treat presbyopia. External ocular lenses, such as spectacles or contact lenses, are typically used to correct vision.

#### What this study adds

- This study demonstrates that UNR844 has the potential to be a first-in-class disease modifying pharmacological therapy for presbyopia, for which there are currently no approved pharmacological treatment interventions. There is a key unmet need for a convenient and safe topical ocular treatment option for presbyopia.

## Supplementary information

Supplementary Material
