# Supplementary material for: Topical lipoic acid choline ester eye drop for improvement of near visual acuity in subjects with presbyopia: a safety and preliminary efficacy trial
Source: Eye (Lond). 2021 Jan 29;35(12):3292–301. doi: 10.1038/s41433-020-01391-z (PMC8602643; doi:10.1038/s41433-020-01391-z)
Supplement: Supplementary file 1 — Supplementary Material [file 41433_2020_1391_MOESM1_ESM.docx]

**Supplementary Materials**

**Study Design**

Overall study design and randomization for the interventional and observational (follow-up) phase of the study are illustrated in Figures S1a and S1b.

**Methods for the supplementary materials**

Inclusion and Exclusion Criteria

Subjects with prior ocular surgery including cataract extraction, intraocular surgery or laser surgery, any history of ocular trauma, a current diagnosis of Type I or uncontrolled Type II diabetes or who took medication known to affect near vision focus or intraocular pressure ≤3 months prior to the study were also excluded. Contact lens use was prohibited for 3 days prior to and for the duration of the study. To be eligible, all female subjects were either post-menopausal or had a negative urine pregnancy test and agreed to the use of an acceptable form of contraception during the study.

Exploratory Endpoint Testing

Cycloplegic refraction was performed at baseline and at the exit visit. Subjective accommodative amplitude testing (defocus curves) was performed, with distance vision measured with an ETDRS chart under controlled lighting conditions and converted to LogMAR scores. Non-dilated pupillary diameter was measured under controlled room illumination using a laminated pupilometer. Subjects were also required to complete a short Near Vision Questionnaire at each follow-up visit and at the exit visit. In a sub-group of subjects at one site, objective accommodative amplitude testing was carried out using ray-tracing wavefront aberrometry (iTrace, Tracey Technologies).

Slit-lamp biomicroscopy assessed the eyelid, conjunctiva, cornea, anterior chamber, and lens, with observations classed as normal or abnormal (grades; minimum [0.5], mild [1.0], moderate [2.0] or severe [3.0]). The dilated fundus exam assessed the vitreous, retina, macula, choroid, and optic nerve at screening and Day 91, and at early termination visits.

**Results for the supplementary materials**

There were no clinically meaningful changes from baseline to Day 91 in the study eye in manifest or cycloplegic refraction (Table S1) or in non-dilated pupil diameter (-0.060mm vs. -0.080mm for UNR844 vs. placebo, respectively; Figure S4).

Defocus curves data demonstrated a trend towards greater accommodative range in the study eye after treatment, supporting DCNVA observations (see manuscript Figures 3 a, b for Day 1-91 data). After dosing ceased, this trend was maintained through month 5 (Day 241; manuscript Figure 3c) and was evident, but declining by month 7 (Day 301; Figure S5).

Examination of the subset of patients with the iTrace Wavefront Aberrometer, using the standard data package provided limited support with this method for measuring accommodation effects of UNR844 Ophthalmic Solution. In the Near Vision Questionnaire, comments from subjects were generally positive and favoured treatment with UNR844 over placebo.

#### Ocular safety endpoints

Acute Comfort Assessments found no clinically significant changes in ocular comfort between the treatment groups. For Day 1 the mean visual analogue scale (VAS) score (SD) was 3.2 (2.4) and 3.3 (2.5) for the UNR844 and placebo groups, respectively. The mean change (SD) in comfort VAS from Day 1 to Day 91 was -1.0 (2.9) for the UNR844 group (n=49) and -1.3 (1.9) for the placebo group (n=23; Table S4), implying a similar modest increase in drug comfort during the course of the study.

#### Distance visual acuity

There were no clinically significant changes in distance visual acuity (BCDVA) in either treatment group compared with baseline measures, with an overall mean change in LogMAR BCDVA (SD) from Day 1 to 91 of -0.022 (0.067) for the UNR844 group and -0.022 (0.064) for the placebo group (Table S4).

#### Intraocular pressure

IOP was within normal range in both treatment groups at baseline and Day 91. There was no clinically significant difference in mean IOP (SD) at baseline between UNR844 [15.2 (2.5) mmHg] and placebo group [14.1 (2.6) mmHg], or in the overall mean change in IOP (SD) from baseline to Day 91, -0.2 (2.5) mmHg for the UNR844 group and 0.0 (2.2) mmHg for the placebo group; Table S4).

#### Slit-lamp biomicroscopy

Minimal or no clinically significant differences were observed in slit-lamp biomicroscopy parameters between the groups or at Day 91 compared with baseline. Small, intermittent changes occurred in both groups with the largest increases occurring in the placebo group. There were no incidents of positive slit-lamp findings categorized as severe in either group. (Table S5)

#### Fundus

There were few differences in the fundus findings (vitreous, retina, macula, choroid and optic nerve) between the study groups and no notable changes during the study. (Table S6)

# Figures and tables

## List of figures

- Figure S1a: Study Design Randomization
- Figure S1b: Study Design Protocol
- Figure S2a: Percent of subjects who experienced a gain of ≥10 letters over time in the **study eye** (Day 1-Day 91) (LOCF)
- Figure S2b: Percent of subjects who experienced a gain of ≥10 letters over time in **bilateral vision (Day 1- Day 91)** (non-LOCF)
- Figure S3: Gains of ≥10 letters in DCNVA (LogMAR) from baseline (Day1) to Day 91, Day 241, and Day 301 in the **study eye**
- Figure S4: Observational Study Cohort [n=52]: Accommodative range defocus curves Day 1 to Day 301 in the **study eye** (non-LOCF)
- Figure S5: Change in pupil diameter (mm) from Day 1 to Day 91 in the **study eye**

## List of tables

- Table S1: DCNVA in LogMAR Change from Baseline – Interventional and Follow-On Observational Studies
- Table S2: **SE, NSE, OU** DCNVA in LogMAR categorical change at Day 91 from Day 1 (non-LOCF) – FAS
- Table S3: Manifest and cycloplegic refraction change Day 1 vs. Day 91 in the **study eye** (FAS; overall)
- Table S4: Acute comfort assessment/Distance visual acuity/IOP by visit Day 1 and Day 91 in the **study eye**
- Table S5: Slit lamp biomicroscopy (% of normal eyes in each group on Day 1 and Day 91 – FAS)
- Table S6: Fundus *data for Visit 1 vs. Day 91 -* ***Study Eye*** *(FAS)*

#### Figure S1a: Study Design Randomization


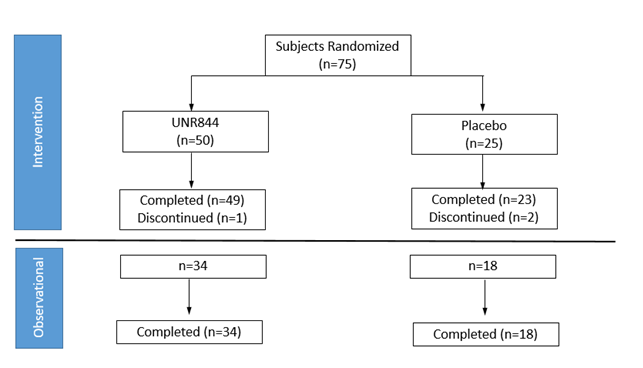


#### Figure S1b: Study Design Protocol

**Day 301**

**Observational**

**(n = 34)**

**Observational**

**(n = 18)**

**UNR844 1.5% BID**

**(n = 50)**

**Placebo BID**

**(n = 25)**

**Day 241**

**Day 91**

**Day 8**

**Day 1**

**Bilateral dosing**

**(both eyes)**

**Post-treatment**

**Unilateral dosing**

**(study eye=non-dominant eye)**

#### Figure S2a: Percent of subjects who experienced a gain of ≥10 letters over time (Day 8 to Day 91) in the **study eye** (LOCF)


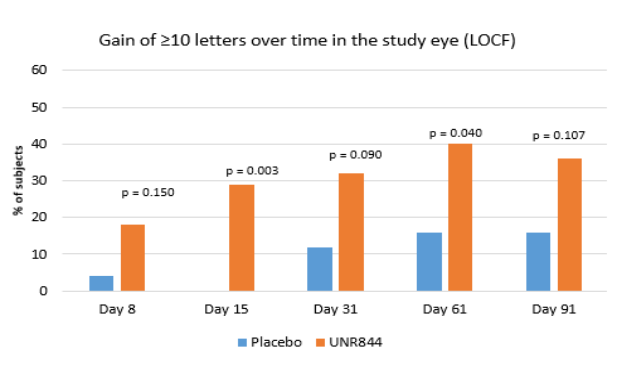


*Figure S2b: Percent of subjects who experienced a gain of ≥10 letters over time (Day 8 to Day 91) in* ***bilateral vision*** *(non-LOCF)*


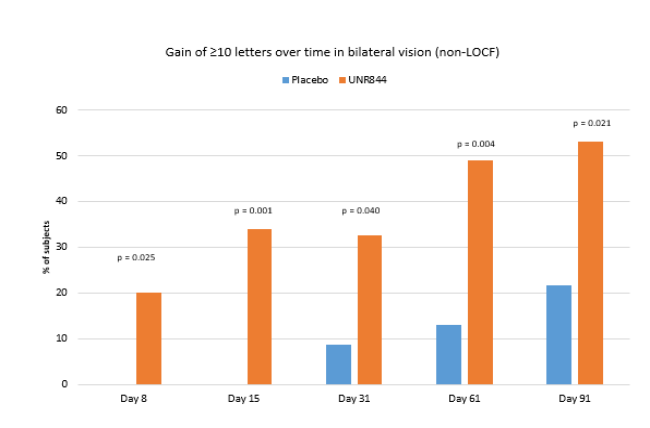


#### Figure S3: Gains of ≥10 letters in DCNVA (LogMAR) from baseline (Day1) to Day 91, Day 241, and Day 301 in the **study eye**

####
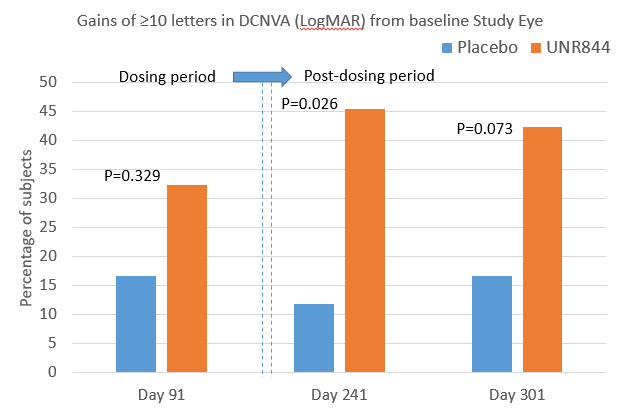


#### Figure S4: Observational Study Cohort [n=52]: Accommodative range defocus curves Day 1 to Day 301 in the **study eye** (non-LOCF)


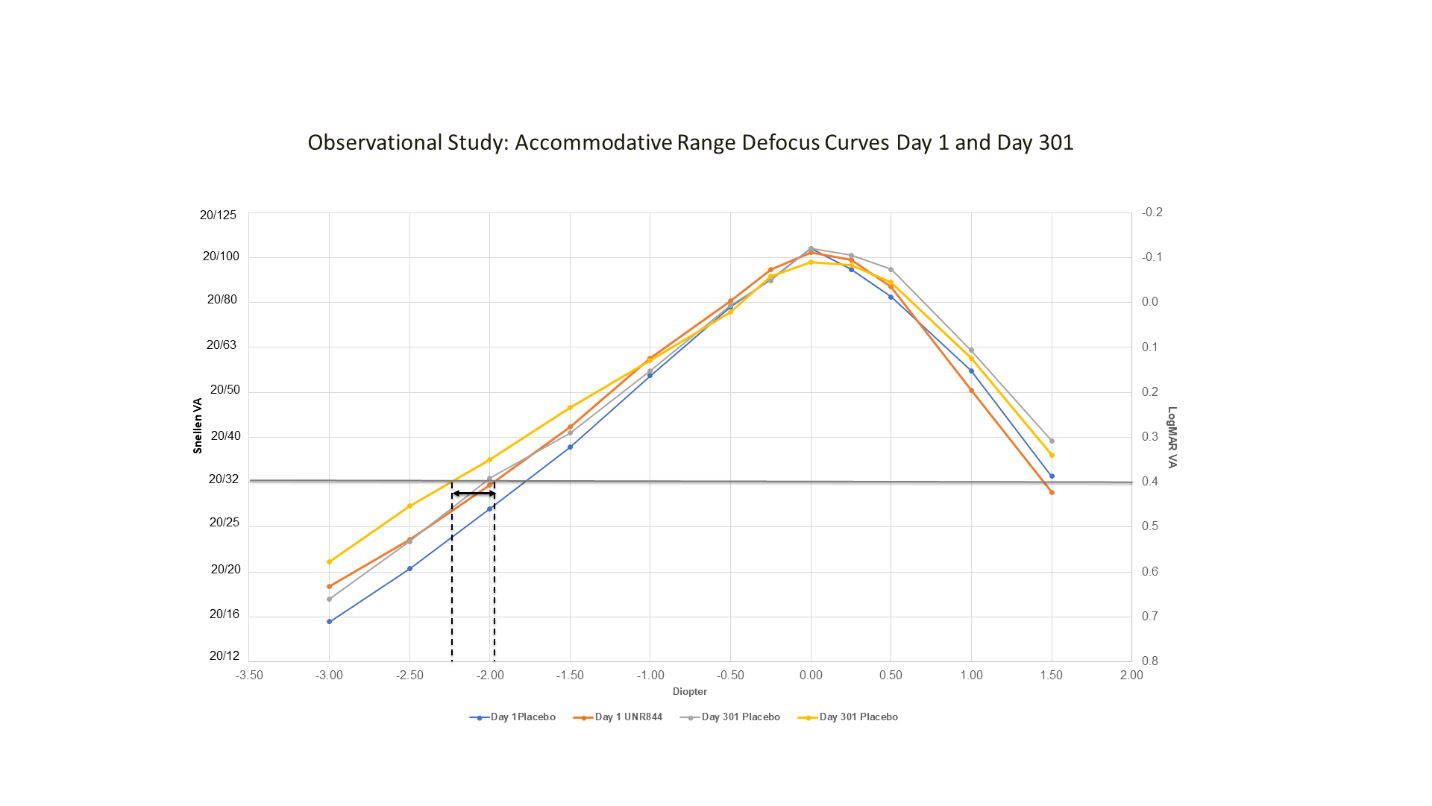


*Figure S5: Change in pupil diameter (mm) from Day 1 to Day 91 in the* ***study eye***


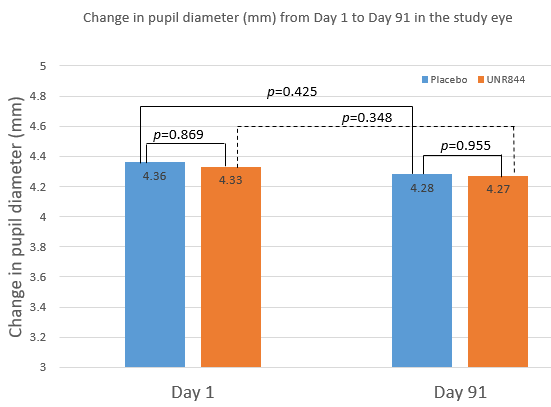


#### Table S1: DCNVA in LogMAR Change from Baseline – Interventional and Follow-On Observational Studies

| **Interventional Study** | | | |
| --- | --- | --- | --- |
| **Overall DCNVA LogMAR Mean Change from Baseline (Day 1) to Day 91 (non-LOCF)** | | | |
|  | **Placebo Ophthalmic Solution**  Mean change from BL (SD) | **UNR844 Ophthalmic Solution**  Mean change from BL(SD) |  |
| **Study Eye***  (non-dominant eye) | -0.086 (0.118) | -0.162 (0.119) | *p* = 0.015 |
| **Non-Study Eye*** | -0.074 (0.128) | -0.160 (0.137) | *p* = 0.012 |
| **Bilateral Vision** – **full cohort*** | -0.089 (0.096) | -0.189 (0.131) | *p* = 0.001 |
| **Bilateral Vision**- Subset BL OU 20/40 or better excluded**^** | -0.099 (0.080) | -0.198 (0.148) | *p* = 0.004 |
|  | | | |
| **Follow-On Observational Study** | | | |
| **Overall DCNVA LogMAR Mean Change from Baseline (Day 1) to Day 91 (non-LOCF)** | | | |
| **Bilateral Vision**  **Follow-on Cohort#** | -0.094 (0.095) | -0.207 (0.130) | *p* < 0.001 |
| **Overall DCNVA LogMAR Mean Change from Baseline (Day 1) to Day 241 (non-LOCF)** | | | |
| **Bilateral Vision**  **Follow-on Cohort#** | -0.051 (0.116) | -0.175 (0.100) | *p* < 0.001 |
| **Overall DCNVA LogMAR Mean Change from Baseline (Day 1) to Day 301 (non-LOCF)** | | | |
| **Bilateral Vision**  **Follow-on Cohort#** | -0.058 (0.186) | -0.165 (0.148) | *p* = 0.043 |
| ***** **Full Cohort** N Day 1: P =25, U=50. Day 91: P = 23, U=49.  **^ BL OU 20/40 subset** **excluded** N Day 1: P = 18, U=35. N Day 91: P = 16, U=34  # **Follow-On Cohort** N Day 91: P=18, U=34. N Day 241 P=17, U=33. N Day 301 P=18, U=33 | | | |

#### Table S2: **SE, NSE, OU** DCNVA in LogMAR categorical change at Day 91 from Day 1 (non-LOCF) – FAS

|  | **Overall** |  | **p-value** |
| --- | --- | --- | --- |
| **Cumulative change category** | **Placebo (n=23)** | **UNR844 (n=49)** | **Placebo vs. UNR844** |
| ***Study eye (non-dominant eye)*** |  |  |  |
| 1 line (≥0.10 LogMAR) | 12 (52.2%) | 35 (71.4%) | 0.121 |
| 2 line (≥0.20 LogMAR) | 4 (17.4%) | 18 (36.7%) | 0.110 |
| 3 line (≥0.30 LogMAR) | 2 (8.7%) | 10 (20.4%) | 0.315 |
| 4 line (≥0.40 LogMAR) | 0 (0.0%) | 1 (2.0%) | 1.000 |
| Any loss (≥0.10 LogMAR) | 0 (0.0%) | 1 (2.0%) | 1.000 |
| No change (-0.90 to 0.90 LogMAR) | 11 (47.8%) | 13 (26.5%) | 0.107 |
| ***Non-study eye*** |  |  |  |
| 1 line (≥0.10 LogMAR) | 11 (47.8%) | 36 (73.6%) | 0.061 |
| 2 line (≥0.20 LogMAR) | 4 (17.4%) | 23 (46.9%) | 0.019 |
| 3 line (≥0.30 LogMAR) | 2 (8.7%) | 9 (18.4%) | 0.484 |
| 4 line (≥0.40 LogMAR) | 0 (0.0%) | 3 (6.1%) | 0.546 |
| Any loss (≥0.10 LogMAR) | 2 (8.7%) | 2 (4.1%) | 0.588 |
| No change (-0.90 to 0.90 LogMAR) | 10 (43.5%) | 11 (22.4%) | 0.095 |
| ***Both eyes*** |  |  |  |
| 1 line (≥0.10 LogMAR) | 12 (52.2%) | 41 (83.7%) | 0.009 |
| 2 line (≥0.20 LogMAR) | 5 (21.7%) | 26 (53.1%) | 0.021 |
| 3 line (≥0.30 LogMAR) | 0 (0.0%) | 11 (22.4%) | 0.013 |
| 4 line (≥0.40 LogMAR) | 0 (0.0%) | 6 (12.2%) | 0.167 |
| Any loss (≥0.10 LogMAR) | 1 (4.3%) | 1 (2.0%) | 0.540 |
| No change (-0.90 to 0.90 LogMAR) | 10 (43.5%) | 7 (14.3%) | 0.015 |

#### Table S3: Manifest and cycloplegic refraction change Day 1 vs. Day 91 in the **study eye** (FAS; overall)

|  | **Manifest refraction** | | **Cycloplegic refraction** | |
| --- | --- | --- | --- | --- |
|  | **Overall** | | **Overall** | |
|  | **Placebo** | **UNR844** | **Placebo** | **UNR844** |
| **Number of subjects randomized** | n=25 | n=50 | n=25 | n=50 |
| **Number of subjects included in the analysis** | n=23 | n=49 | n=23 | n=49 |
| **Sphere (D)** |  |  |  |  |
| Mean | -0.011 | 0.031 | -0.033 | 0.005 |
| SD | 0.232 | 0.345 | 0.314 | 0.380 |
| Min, Max | -0.25, 0.50 | -1.25, 1.00 | -0.75, 0.75 | -0.75, 1.25 |
| **Cylinder (D)**  **(minus cylinder)** |  |  |  |  |
| Mean | -0.022 | -0.005 | -0.065 | 0.001 |
| SD | 0.198 | 0.201 | 0.274 | 0.265 |
| Min, Max | -0.50, 0.50 | -0.50, 0.50 | -0.75, 0.50 | -0.50, 0.75 |
| **Axis (degrees)** |  |  |  |  |
| Mean | -7.783 | 7.020 | -9.174 | -2.490 |
| SD | 38.818 | 55.299 | 33.809 | 60.288 |
| Min, Max | -175.0, 25.0 | -110.0, 170.0 | -153.0, 20.00 | -180.0, 180.0 |
| **Spherical equivalent** |  |  |  |  |
| Mean | -0.022 | 0.028 | -0.065 | 0.005 |
| SD | 0.202 | 0.311 | 0.235 | 0.364 |
| Min, Max | -0.38, 0.38 | -1.00.1.00 | -0.50, 0.38 | -0.75, 1.25 |

#### Table S4: Acute comfort assessment/Distance visual acuity/IOP by visit Day 1 and Day 91 in the study eye

|  | **Acute comfort assessment**  Scale 0-10  (0 more comfortable) | | **Distance visual acuity**  BCDVA LogMAR | | **Intraocular pressure***  mmHg | |
| --- | --- | --- | --- | --- | --- | --- |
|  | **Placebo** | **UNR844** | **Placebo** | **UNR844** | **Placebo** | **UNR844** |
| **Day 1 (baseline)**  ***Visit 1 (serves as baseline for IOP)** |  |  |  |  |  |  |
| **N** | 25 | 50 | 25 | 50 | 25 | 50 |
| **Mean** | **3.3** | **3.2** | **-0.109** | **-0.118** | **14.1** | **15.2** |
| **SD** | 2.5 | 2.4 | 0.075 | 0.063 | 2.6 | 2.5 |
| **Day 91** |  |  |  |  |  |  |
| **N** | 23 | 49 | 23 | 49 | 23 | 49 |
| **Mean** | **2.1** | **2.1** | **-0.136** | **-0.136** | **14.1** | **14.9** |
| **SD** | 2.2 | 2.4 | 0.080 | 0.080 | 2.5 | 2.9 |
| **Change from Day 1 to Day 91** |  |  |  |  |  |  |
| **N** | 23 | 49 | 23 | 49 | 23 | 49 |
| **Mean** | **-1.3** | **-1.0** | **-0.022** | **-0.022** | **0.0** | **-0.2** |
| **SD** | 1.9 | 2.9 | 0.064 | 0.067 | 2.2 | 2.5 |

#### Table S5: Slit lamp biomicroscopy (% of normal eyes in each group on Day 1 and Day 91 – FAS)

|  | **Day 1** |  | **Day 91** |  |
| --- | --- | --- | --- | --- |
|  | **Placebo (n=25)** | **UNR844 (n=50)** | **Placebo (n=25)** | **UNR844 (n=50)** |
| **Eyelid** | 76.0 | 94.0 | 68.0 | 92.0 |
| **Conjunctiva** | 60.0 | 80.0 | 52.0 | 78.0 |
| **Cornea (epi)** | 100.0 | 96.0 | 92.0 | 94.0 |
| **Cornea (stroma)** | 100.0 | 96.0 | 84.0 | 94.0 |
| **Cornea (endo)** | 100.0 | 98.0 | 92.0 | 96.0 |
| **Anterior chamber (cells)** | 100.0 | 100.0 | 92.0 | 98.0 |
| **Anterior chamber (flare)** | 100.0 | 100.0 | 92.0 | 98.0 |
| **Anterior chamber (sclera)** | 100.0 | 100.0 | 92.0 | 98.0 |
| **Lens (ant capsule)** | 100.0 | 100.0 | 88.0 | 98.0 |
| **Lens (post capsule** | 100.0 | 100.0 | 92.0 | 98.0 |
| **Lens (lens)** | 80.0 | 86.0 | 72.0 | 84.0 |
| **Lens (nucleus)** | 76.0 | 86.0 | 68.0 | 84.0 |

*Table S6 Fundus Data for Visit 1 vs. Day 91 - Study Eye (FAS)*

|  | **Overall Visit 1** | | **Overall Day 91** | |
| --- | --- | --- | --- | --- |
|  | **Placebo (N=25)** | **UNR844  (N=50)** | **Placebo (N=25)** | **UNR844**  **(N=50)** |
| **Vitreous**  **Study Eye** |  |  |  |  |
| **Normal** | **23 (92.0%)** | **49 (98.0%)** | **22 (88.0%)*** | **49 (98.0%)*** |
| **Abnormal** | **2 (8.0%)** | **1 (2.0%)** | **1 (4.0%)** | **0 (0%)** |
| Minimal | 0 (0%) | 1 (2.0%) | 1 (4.0%) | 0 (0%) |
| Mild | 2 (8.0%) | 0 (0%) |  |  |
| **Retina**  **Study Eye** |  |  |  |  |
| **Normal** | **22 (88.0%)** | **49 (98.0%)** | **20 (80.0%)*** | **48 (96.0%)*** |
| **Abnormal** | **3 (12.0%)** | **1 (2.0%)** | **3 (12.0%)** | **1 (2.0%)** |
| Minimal | 1 (4.0%) | 0 (0%) | 1 (4.0%) | 0 (0%) |
| Mild | 2 (8.0%) | 1 (2.0%) | 2 (8.0%) | 1 (2.0%) |
| **Macula**  **Study Eye** |  |  |  |  |
| **Normal** | **25 (100.0%)** | **50 (100.0%)** | **23 (92.0%)*** | **49 (98.0%)*** |
| **Abnormal** | **0 (0%)** | **0 (0%)** | **0 (0%)** | **0 (0%)** |
| **Choroid**  **Study Eye** |  |  |  |  |
| **Normal** | **25 (100.0%)** | **50 (100.0%)** | **23 (92.0%)*** | **49 (98.0%)*** |
| **Abnormal** | **0 (0%)** | **0 (0%)** | **0 (0%)** | **0 (0%)** |
| **Optic Nerve**  **Study Eye** |  |  |  |  |
| **Normal** | **19 (76.0%)** | **48 (96.0%)** | **18 (72.0%)*** | **47 (94.0%)*** |
| **Abnormal** | **6 (24.0%)** | **2 (4.0%)** | **5 (20.0%)** | **2 (4.0%)** |
| Minimal | 1 (4.0%) | 0 (0%) | 1 (4.0%) | 0 (0%) |
| Mild | 5 (20.0%) | 2 (4.0%) | 4 (16.0%) | 2 (4.0%) |

* The % of subjects calculation is based on all subjects who received study product, the FAS by treatment group N, a denominator of Placebo N=25 and UNR844 N=50 (Visit 1).
